# Prenatal cadmium exposure has inter-generational adverse effects on Sertoli cells through the follicle-stimulating hormone receptor pathway

**DOI:** 10.1530/REP-23-0070

**Published:** 2023-09-04

**Authors:** Xiaoqin Li, Zhilan Lu, Xiushuai Du, Youbin Ye, Jianlin Zhu, Yuchen Li, Jin Liu, Wenchang Zhang

**Affiliations:** 1 Department of Preventive Medicine, Fujian Provincial Key Laboratory of Environmental Factors and Cancer, Key Laboratory of Environment and Health, School of Public Health, Fujian Medical University, Fuzhou, China

## Abstract

**In brief:**

Exposure to cadmium (Cd) during pregnancy can potentially harm the reproductive system of male offspring. This article shows that pregnant woman should be protected from cadmium exposure.

**Abstract:**

Exposure to cadmium (Cd) during pregnancy can potentially harm the reproductive system of male offspring, although the full extent of its heritable effects remains partially unresolved. In this study, we examined the inter-generational impacts of Cd using a distinct male-lineage generational model. Pregnant Sprague–Dawley female rats (F0) were administered control or cadmium chloride (0.5, 1 and 2 mg/day) via intra-gastric administration from gestation day 1 to 20. Subsequently, the first filial generation (F1) male rats were mated with untreated females (not exposed to Cd) to produce the second filial generation (F2). Histopathological analysis of the F1 and F2 generations revealed abnormal testicular development, while ultrastructural examination indicated damage to Sertoli cells. Cd exposure also led to alterations in serum hormone levels (gonadotropin-releasing hormone, follicle-stimulating hormone) and reduced follicle-stimulating hormone receptor (FSHR) protein expression in Sertoli cells in the F1 generation. Furthermore, Cd affected the mRNA and protein expression of FSHR pathway factors and DNA methyltransferase, albeit with distinct patterns and inconsistencies observed between the F1 and F2 generations. Overall, our findings indicate that prenatal Cd exposure, using a male-lineage transmission model, can induce inter-generational effects on male reproduction, particularly by causing toxicity in Sertoli cells. This effect appears to be primarily mediated through disruptions in the FSHR pathway and changes in DNA methyltransferase activity in the male testes.

## Introduction

Cadmium (Cd) is a potentially toxic metal at trace levels that is widely present in the environment due to industrial and agricultural applications (Quraishi *et al.* 2016). Intrauterine exposure to Cd can reduce the wet weight of testes of male offspring rats, change the histology of the testis, cause a significant decrease in daily sperm production and then influence the quality of epididymal sperm (Chemek *et al.* 2018). At the same time, studies have shown that prenatal Cd exposure during pregnancy inhibits the development of testicular Leydig cells in adult offspring (first filial generation, F1) rats, as well as the activation of the cAMP/protein kinase A signalling pathway (Tian *et al.* 2018). Cd exposure also down-regulates the expression of steroid-producing enzymes (Tian *et al.* 2018). These results indicate that Cd exposure has an impact on the reproductive health of male offspring.

Sertoli cells are one of the main cells in the testis and provide protection and nutrition for spermatogenesis. A lack of Sertoli cells or an insufficient number of Sertoli cells may lead to degeneration, apoptosis and necrosis of spermatogenic cells and male infertility (Leal & França 2009, Papaioannou *et al.* 2009). The number of Sertoli cells depends on their proliferation ability, and many factors affect proliferation, such as follicle-stimulating hormone (FSH), insulin family growth factors, transforming growth factor super family, androgens, oestrogen and cytokines (Meroni *et al.* 2019). Among them, FSH plays a major regulatory role in the growth and development of Sertoli cells. Studies have found that when FSH binds to follicle-stimulating hormone receptor (FSHR), it increases transcriptional activity to activate different signalling pathways, and phosphorylating downstream proteins affects Sertoli cell proliferation (Gloaguen *et al.* 2011, Ulloa-Aguirre *et al.* 2018). The PI3K/AKT pathway is the main target for the effect of chemicals on the proliferation of Sertoli cells (Meroni *et al.* 2019). Nitrofurazone can activate the PI3K/AKT/FOXO1/TNF-α pathway to produce toxic effects on mouse sperm and testicular tissue (Li *et al.* 2019). *In vitro* exposure to nitrofurazone in primary Sertoli cells also obtained the same results (Li *et al.* 2019). However, few studies have focused on whether prenatal Cd exposure causes FSHR pathway problems in an inter-generational manner and leads to reproductive toxicity in Sertoli cells of male offspring at different developmental stages (post-natal day (PND) 5, PND 21 and PND 56). Therefore, our study aims to investigate whether prenatal Cd exposure during pregnancy interferes with FSH secretion in male offspring, affects the expression of FSHR in Sertoli cells and then changes the FSHR/PI3K/AKT/FOXO1 signalling pathway, resulting in inhibition of the proliferation of Sertoli cells and ultimately affecting the health of male offspring.

Epigenetic studies have shown (Baccarelli & Bollati 2009) that Cd can reduce genome methylation and inhibit DNA methyltransferase in a noncompetitive manner, and after high-dose exposure to Cd, the methylation status of the entire genome and DNA methyltransferase (DNMT3A/3B) mRNA expression levels of the testis were higher (Nakayama *et al.* 2019). Studies also (Castillo *et al.* 2012) found that the expression of DNA methyltransferases (DNMT3A and DNMT3b or only DNMT3A) was related to DNA hypomethylation in animals exposed to extremely high doses of Cd. Whether Cd can interfere with the DNA methylation patterns of offspring testes by affecting DNA methyltransferases to cause reproductive damage is worthy of further study.

More importantly, in this study, the prenatal Cd exposure dose we used was based on an average residential Cd exposure of 0.1889 mg/kg. Based on this, human equivalent dose (HED) (mg/kg) = animal dose (mg/kg) × (animal km/human km) (Nair *et al.* 2018) was calculated according to the estimated human dose of Cd exposure and the equivalent dose of human and mouse body surface area. Cd exposure not only affects the reproductive health of the males of the growth and development of the offspring but also has a profound impact on the adult reproductive health of the subsequent offspring, with inter-generational reproductive toxicity effects. In the current situation of low fertility and worsening environmental pollutants, it is necessary to prevent and control Cd exposure during pregnancy. Accordingly, this study provides an important scientific basis for controlling Cd.

To investigate how prenatal Cd exposure in female rats (F0) during gestation may affect the reproductive development of their male offspring, we studied serum hormone, testicular tissue, FSHR and its pathway and DNA methyltransferases in the first (F1), and second (F2) filial generations following F0 female rat exposure to the metal. The mechanism leading to the early onset of puberty of the offspring induced by such exposure was explored. These studies might provide more insight and a better understanding of the inter-generational effect and epigenetic inheritance in humans by environmental metal pollution.

## Materials and methods

### Experimental design

Sprague–Dawley (SD) rats (20 males, 220–250 g, and 32 females, 250–280 g) were purchased from the laboratory centre of Fujian Medical University (No 2018-12DM093) and housed together overnight at a male-to-female ratio of 1 : 2. The animal compartment was kept on a 14 h light:10 h darkness photoperiod at a controlled temperature of 20 ± 2℃) and relative humidity of 70 ± 10%.

After mating, the females were checked the next morning, and the presence of a vaginal plug was considered gestational day (GD) 0. These pairs were the original generation (F0). The pregnant rats were divided into four groups (8 rats per group), and three different concentrations of Cd exposure (0.5, 1 and 2 mg/kg/day of CdCl_2_ dissolved in ultrapure water) and control (ultrapure water, Merck Millipore). CdCl_2_ was purchased from Sigma-Aldrich. The concentrations were chosen based on previous studies and their environmental relevance: in China, the average dose of Cd in rice alone is approximately 0.05–0.12 mg/kg (Wang *et al.* 2019). Hence, the Cd dosage groups in this study were selected based on the estimated human exposure and the equivalent dose ratio calculated from the surface area of humans and rats (HED (mg/kg) = animal dose (mg/kg) × (animal km/human km).

Each pregnant rat was treated with intra-gastric administration daily throughout pregnancy and allowed to deliver naturally. The day of birth was considered PND 0. Rats born from the F0 generation were labelled F1generation. The F1 males were mated fertility-confirmed, untreated females to create a F2 filial generation (Supplementary Fig. 1, see section on supplementary materials given at the end of this article). The male F1 and F2 rats were routinely fed until PND 5, 21 and 56 (these three key stages of testicular cell growth and development), then sacrificed to obtain testes and blood. All animal experiments were performed with proper care in accordance with the Guide for the Care and Use of Laboratory Animals at Fujian Medical University.

The proliferation of Sertoli cells has a specific period, such as in rats, where the proliferation of testicular Sertoli cells mainly occurs during the foetal period, 15 days after birth and early puberty, with a fixed number of Sertoli cells during other periods. Based on our research, we chose the PND 5, 21 and 56 to study.

There were ten males in F1 generations from each group respectively to generate the F2 generation. Then 20 male testes were collected in each group at both two generations for the designed experiments.

### Histological and morphology examinations

#### Light microscopy

Testes were collected from two generations, then fixed in Bouin’s fluid (BBI Life Sciences, Shanghai, China) and embedded in paraffin wax. Serial sections were cut at a 5 μm thickness and stained with haematoxylin and eosin (H&E). These steps have been described by Lopes *et al.* 2016.

Five testes in each group from F1 and F2 rats were used for histological examination, and five slices taken from 25 serial slices of each testis were used for morphological examination. In each slice, 15 seminiferous tubules (STs) were noted (after H&E staining, five fields of view were selected, and three sections of STs were then selected in each field of view) for the Johnsen score (Glander *et al.* 2000). Within these same slices, the diameter of every ST and Johnsen score in Table 1 (PND 56) was also measured.

**Table 1:** Johnsen score.

Score	Explanation
10	Full spermatogenesis
9	Many late spermatids, disorganised tubular epithelium
8	Few late spermatids
7	No late spermatids, many early spermatids
6	No late spermatids, few early spermatids, arrest of spermatogenesis at the spermatid stage, disturbance of spermatid differentiation
5	No spermatids, many spermatocytes
4	No spermatids, few spermatocytes, arrest of spermatogenesis at the primary spermatocyte stage
3	Spermatogonia only
2	No germ cells, Sertoli cells only
1	No seminiferous epithelial cells, tubular sclerosis

#### Transmission electron microscopy (TEM)

Two testes each for the control group and 2 mg/kg group at PND 5 and PND 56 were cut into pieces (1 mm (mm) × 1 mm × 1 mm) for transmission electron microscopy (TEM) from F1 generation; the pieces were washed, post-fixed and dehydrated. Then, 100 nm (nm) sections were cut (Leica) and stained with uranyl acetate and alkaline lead citrate and visualised under a transmission electron microscope (Quanta 450, Hillsborough, OR, USA).

### Immunohistochemistry staining

Five testes for the control group and 2 mg CdCl_2_/kg group from PND 56 of F1 and F2 were fixed in 10% formaldehyde solution, embedded in paraffin wax and then serially sectioned into 5 μm thick slices. Subsequently, the sections were deparaffinised, added 3% (w/w) H_2_O_2_ for 10 min, rehydrated (PBS, three times, 5 min each), microwaved (high setting, six times, 5 min each) and water-bath heated (95℃, 20 min) in citrate buffer (pH 6.0). The sections were blocked with blocking solution (goat serum) for 1 h at room temperature before being incubated with an anti-FSHR antibody (diluted 1 : 50; catalogue number: BD-PT1795, Biodragon, China,) for 12 h at 4℃. Subsequently, after rinsing the sections thoroughly with PBS, they were incubated with a biotinylated secondary antibody in an antibody solution (goat serum) for 1 h at room temperature before being stained with DAB and then counterstained. AOD means average optical density. IOD means integrated option density. Calculated the AOD value according to AOD = IOD/area to count the expression of FSHR protein.

### The determination of hormones in serum

Whole blood was collected from rats (nine rats were from F1 and F2 in each group) and centrifuged at 7104 ***g*** for 10 min at 4 °C, and serum was used for the determination of hormones (gonadotropin-releasing hormone (GnRH) and follicle-stimulating hormone (FSH)). We used enzyme-linked immunosorbent assay (ELISA) kits (Elabscience Biotechnology, Wuhan, China; Number: E-EL-0071c, E-EL-R0391c) to measure the concentrations of these hormones on a microplate reader (wavelength of 450 nm).

### Semi-quantitative real-time PCR analysis

Total RNA was extracted from the testes in two generations (ten testes from PND 5, five testes from PND 21 and 56) with TRIzol reagent (catalogue number: 15596018, Invitrogen), and the concentration and purity of the samples were determined by measuring the 260 nm : 280 nm absorbance ratio using an Ultra-micro UV-Vis spectrophotometer (Denovix, Wilmington, DE, USA). cDNA was obtained from 1μg RNA by reverse transcription using a Prime Script RT reagent kit (catalogue number: RR047A, Takara Biotechnology, Dalian, China). PCR amplification was performed with the SYBR Green I fluorochrome (catalogue number: RR420A, SYBR® Premix Ex Taq™, Takara Biotechnology) and a Light Cycler 480 System (Roche). Each reaction contained 10 μL of SYBR Premix Ex Taq, 0.8 μL of forward and reversed primer (10 μM), 2 μL of cDNA and RNase-free dH_2_O to 20 μL. The primers were synthesised by Sangong Biological Technology (Shanghai, China), the sequences of which are shown in Table 2. After the reaction, the fluorescence signal reaction data was collected and stored by the Roche LightCycler480 PCR Amplifier. The melting curve conditions: 95℃ for 5 s; 60℃ for 1 min; 95℃ for 15 s, 1 cycle. The melting curve determines the amplified product, specificity and analysis. Set three replicate holes for each sample and each gene. The purity of each sample is within the normal range of 1.8–2.1. Therefore, to correct the initial total DNA amount, the 2^−ΔΔCt^ method of Livak & Schmittgen (2001) was used in the study to calculate the mRNA expression of each gene.

**Table 2:** Sequences of primers used for real-time RT-PCR amplification.

Target gene	Primer sequences (5′ → 3′)	
	Forward	Reverse
*Gapdh*	GTTACCAGGCTGCCTTCTC	GATGGTGATGGGTTTCCCGT
*Fshr*	CGTCATGGTATTGGGCTGGA	GCAGGCAGATGCTCACTTTCA
*Pi3k*	ATCCATTAACCTTGGGCTGGA	GTAGAGCAACTTCACATC
*Akt*	CTAGGATGATGGAGGTAG	CCGAGGAGTTTGAGATAA
*Foxo1*	CAGCCAGGCACCTCATAACA	TCAAGCGGTTCATGGCAGAT
*Dnmt1*	GGTACAGCCAGGACTACGCAAG	TCAGCCAGCCGGATACAAGA
*Dnmt3a*	CCCGAAGGTTTACCCACCTG	GCGATGTAGCGGTCCACTTG
*Dnmt3b*	TGGCAAGGATGACGTTCTGTG	GCAAACAGGTGTCTGATGACTGG

### Western blot analysis

Total protein was extracted from the testes in two generations (ten testes from PND 5, five testes from PND 21/56) using RIPA lysis solution (catalogue number: P0013B, Beyotime, Shanghai, China). The protein concentrations were measured using a BCA protein assay kit (catalogue number: P0012S, Beyotime, Shanghai, China). Then, the proteins were separated by SDS-PAGE with a 4% stacking gel and a 10% separating gel for 30 min at 80 V and 1 h at 100 V, respectively, and then transferred onto polyvinylidene fluoride membranes. After blocking the membranes at 4℃ overnight in TBST buffer containing 5% milk, they were incubated with specific primary antibodies for 18 h at 4℃ (Table 3). Then, after being washed three times in TBST, the membranes were incubated with secondary antibodies conjugated to goat anti-rabbit IgG (H + L)/HRP (1 : 5000, catalogue number: bs-40295G-HRP, BIOSS, Beijing, China). ECL Plus Detection Reagent (catalogue number: P1010, Applygen Technologies, Beijing, China) was used for membrane exposure. The values were normalised to the corresponding GAPDH levels and then expressed as a percentage of the control value using ImageJ (protein imaging using Tanon 5200, Shanghai, China).

**Table 3:** Antibodies and conditions for western blotting.

Protein	Source	Dilution	Manufacturer	Catalogue No.
GAPDH	Rabbit	1:10,000	Abcam	ab181602
FSHR	Rabbit	1:5000	Invitrogen	PA5-99424
FSHR	Rabbit	1:50	Biodragon, China	BD-PT1795
PI3K	Rabbit	1:1000	Abcam	ab109006
AKT	Rabbit	1:10,000	Abcam	ab179463
FOXO1	Rabbit	1:10,000	CST, UK	2880S
DNMT1	Rabbit	1:2000	ABclonal, China	A16729
DNMT3A	Rabbit	1:2000	ABclonal, China	A16834
DNMT3B	Rabbit	1:1000	ABclonal, China	A2899

### Single-gene DNA methylation analyses

#### DNA extraction

Genomic DNA was extracted from three different testes in F1 and F2 generations at PND 56 in control and 2 mg CdCl_2_/kg groups with the Blood and Tissue DNA Kit (QIAGEN) according to the manufacturer’s instructions. The concentration and purity of the DNA were determined by absorbance at 260 and 280 nm.

#### Bisulphite treatment

A total of 200 ng genomic DNA from each sample was bisulphite-treated with the Methylamp DNA Modification Kit (Epigentek). The quality of the bisulphite conversion was controlled by using PCR products that had no methyl group. Sequencing results confirmed that 96.6% of cytosine residues were converted.

#### Fshr, Akt and Foxo1 methylation analyses

The Sequenom MassARRAY platform (CapitalBio, Beijing, China) was used to perform the quantitative methylation analysis. The system used matrix-assisted laser desorption/ionisation time-of-flight (MALDI-TOF) mass spectrometry in combination with RNA base-specific cleavage (MassCLEAVE). A detectable pattern was then analysed for its methylation status. PCR primers were designed with Methprimer (http://epidesigner.com) through the promoter regions of target genes. The target regions were amplified using the primer pairs shown in Table 4. Each forward primer was tagged with a 10-mer (5′-AGGAAGAGAG-3′) to balance the PCR, and each reverse primer had a T7-promoter tag (5′-CAGTAATACGACTCACTATAGGG AGAAGGCT-3′) for *in vitro* transcription. Polymerase chain reaction amplification was performed with the following parameters: hot start at 94℃ for 15 min, followed by denaturing at 94℃ for 20 s, annealing at 56℃ for 30 s, extension at 72℃ for 1 min for 45 cycles and final incubation at 72℃ for 3 min. Unincorporated dNTPs were dephosphorylated by adding 2 mL of premix including 0.3 U shrimp alkaline phosphate (SAP; Sequenom, San Diego, CA, USA). The reaction mixture was incubated at 37℃ for 40 min, and SAP was then heat-inactivated for 5 min at 85℃. After SAP treatment, 2 mL of the PCR products were used as a template for *in vitro* transcription, and RNase A cleavage was used for the reverse reaction, following the manufacturer’s instructions. The samples were conditioned and spotted on a 384-pad Spectro-CHIP using a MassARRAY nano-dispenser (Samsung, Irvine, CA, USA), followed by spectral acquisition on a MassARRAY Compact analyser. The resultant methylation calls were analysed with EpiTyper software v1.0 to generate quantitative results for each CpG site or an aggregate of multiple CpG sites.

**Table 4:** Synthetic oligonucleotides used in the study.

	Primer sequence
*Fshr* promoter sequencing primers	
*Fshr*-1L	TTTTTGTGTATTATGTTATGAAGAATTG
*Fshr*-1R	AAAAACAAAAACAAACCATTCATTT
*Fshr*-5L	TGGGTTGAAGGTAGAAAGTTTTTTT
*Fshr*-5R	TCACATTATTTTACTACACAAAAATCCC
*Fshr*-8L	TTTTTTTGTTATTTTGGGGGTTAAG
*Fshr*-8R	CAAATAAAAACCACACAACTATCCC
*Akt* promoter sequencing primers	
*Akt*-1L	TTTTGGGAAGGAGAATTTATTTTTT
*Akt*-1R	AATCAACCCCTCTAAAACACTAACC
*Akt*-9L	TTGGGTTAGGGAGAAGAGATATAGG
*Akt*-9R	AATACTCCTCAAACAAAACCCCTAC
*Akt*-33L	AAGAGGTTTTAGGAGGAGGTTATGA
*Akt*-33R	TTAAAAACCAAAAAACCAAAAACAA
*Foxo1* promoter sequencing primers	
*Foxo1*-12L	GGTTTGTAGGTGTGTATAGGTAGGG
*Foxo1*-12R	AAATATTTTTCTTTCTCACACACTTTTT
*Foxo1*-14L	TATTGGTTGTTTAGGTAGAGATTTGG
*Foxo1*-14R	CCACCAACAAAAAATACCAAAAA
*Foxo1*-15L	GGTATTTTTTGTTGGTGGGGG
*Foxo1*-15R	TCTCTTCTAACAAACTCAAATTACTCA
*Foxo1*-17L	TGAGTAATTTGAGTTTGTTAGAAGAGA
*Foxo1*-17R	CCCTTATCCTTAAAATAAAACACACTC
10mer-tagged or T7-tagged primers	
*Fshr*-1L	aggaagagagTTTTTGTGTATTATGTTATGAAGAATTG
*Fshr*-1R	cagtaatacgactcactatagggagaaggctAAAAACAAAAACAAACCATTCATTT
*Fshr*-5L	aggaagagagTGGGTTGAAGGTAGAAAGTTTTTTT
*Fshr*-5R	cagtaatacgactcactatagggagaaggctTCACATTATTTTACTACACAAAAATCCC
*Fshr*-8L	aggaagagagTTTTTTTGTTATTTTGGGGGTTAAG
*Fshr*-8R	cagtaatacgactcactatagggagaaggctTCACATTATTTTACTACACAAAAATCCC
*Akt*-1L	aggaagagagTTTTGGGAAGGAGAATTTATTTTTT
*Akt*-1R	cagtaatacgactcactatagggagaaggctAATCAACCCCTCTAAAACACTAACC
*Akt*-9L	aggaagagagTTGGGTTAGGGAGAAGAGATATAGG
*Akt*-9R	cagtaatacgactcactatagggagaaggctAATACTCCTCAAACAAAACCCCTAC
*Akt*-33L	aggaagagagAAGAGGTTTTAGGAGGAGGTTATGA
*Akt*-33R	cagtaatacgactcactatagggagaaggctTTAAAAACCAAAAAACCAAAAACAA
*Foxo1*-12L	aggaagagagGGTTTGTAGGTGTGTATAGGTAGGG
*Foxo1*-12R	cagtaatacgactcactatagggagaaggctAAATATTTTTCTTTCTCACACACTTTTT
*Foxo1*-14L	aggaagagagTATTGGTTGTTTAGGTAGAGATTTGG
*Foxo1*-14R	cagtaatacgactcactatagggagaaggctCCACCAACAAAAAATACCAAAAA
*Foxo1*-15L	aggaagagagGGTATTTTTTGTTGGTGGGGG
*Foxo1*-15R	cagtaatacgactcactatagggagaaggctTCTCTTCTAACAAACTCAAATTACTCA
*Foxo1*-17L	aggaagagagTGAGTAATTTGAGTTTGTTAGAAGAGA
*Foxo1*-17R	cagtaatacgactcactatagggagaaggctCCCTTATCCTTAAAATAAAACACACTC

### Statistical analysis

The experimental data used SPSS version 23.0, for statistical analysis. Quantitative data underwent a normality test and variance homogeneity test. Then the data were processed as follows: in line with normal distribution and variance homogeneity, a one-way ANOVA was performed, and a two-sample *t*-test was used between the two samples. For other quantitative data results, Dunnett’s *t*-test was used to compare all the infected groups with the control group; if the normal distribution or homogeneity of variance was not met, the rank-sum test (Kruskal–Wallis test) was considered. All of the data are expressed as mean ± s.d. or number. Differences were considered significant at *P* < 0.05 or *P* < 0.01.

## Results

### Prenatal Cd exposure disrupted testicular development in F1 and F2 generations and Sertoli cells may be the main target cells

**Figure 1:**
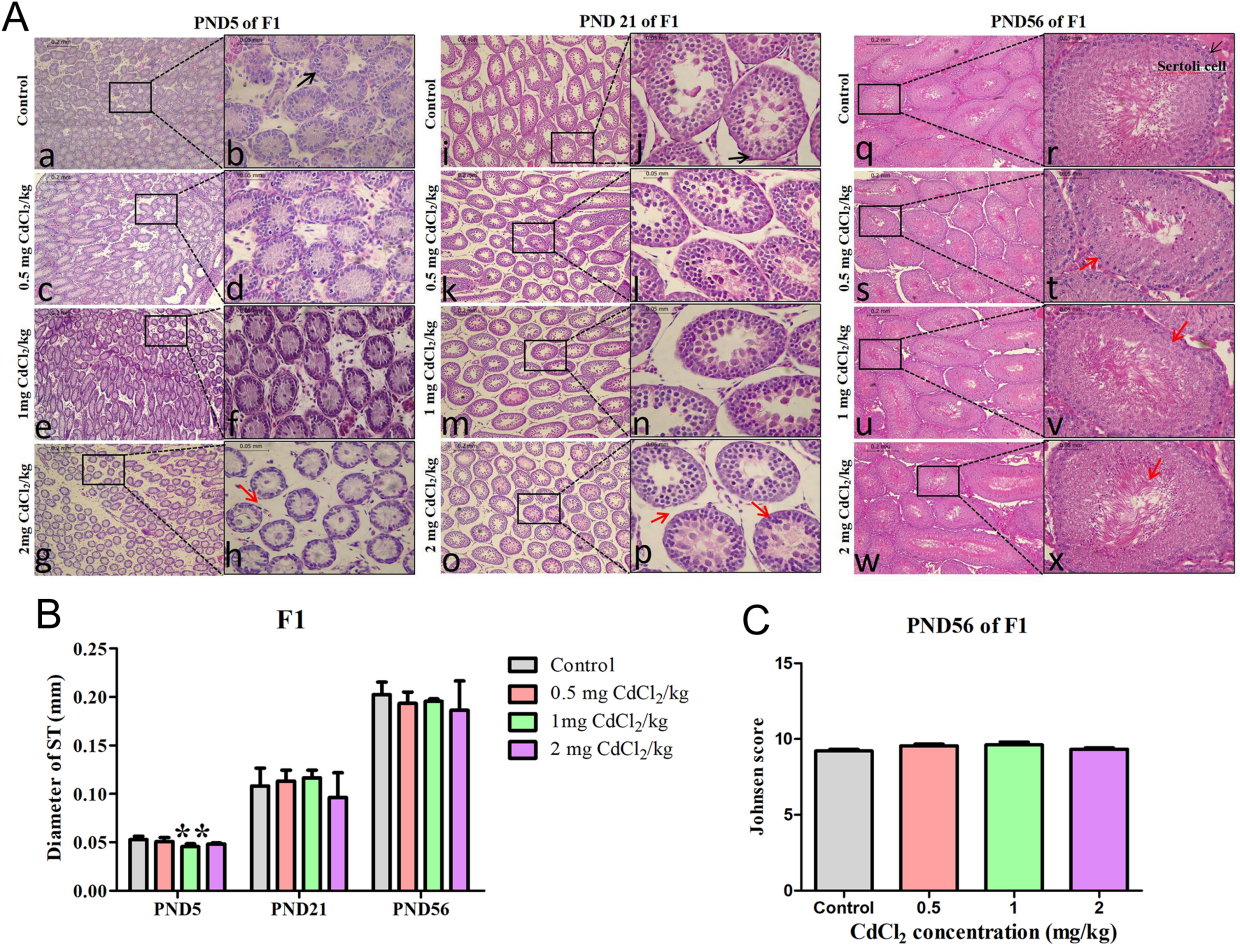
Effect of prenatal Cd exposure on testicular histology in the F1 generation rats. A: H&E staining at different stages of F1 generation after Cd exposure to Cd during pregnancy. B: The tubular diameter (ST diameter of 375 tubules) of F1. C: The Johnsen score of PND 56 of F1. *n* = 5 rats per group. Data are presented as mean ± s.d., **P < 0.01 compared with the control group. Black arrow indicates immature Sertoli cell, whilst red arrow indicates damaged structure.

By the light microscope, the histological examination in the control group found that STs had normal morphology, immature Sertoli cells were seen around the STs and a layer of spermatogonia was found in each ST at PND 5 (black arrow in Fig. 1Ab); also, all the cells were getting closely connected, and immature Sertoli cells also can be seen in the STs at PND 21 (black arrow in Fig. 1Aj); until PND 56, all the cells seen in the STs had a normal shape, and mature Sertoli cells were seen around the STs and layers of spermatogonia were found in each ST (Fig. 1Ar).

However, compared to the control group, some changes were observed in the treatment groups in the F1 generation. The diameter of ST in the 1 mg CdCl_2_/kg-treated group significantly decreased at PND 5 (*P* < 0.05, Fig. 1B), and the arrangement of spermatogenic cells can be seen as partially disorderly at PND 56 (red arrow in Fig. 1Ax). At the same time, compared with the control group, the Johnsen score has no statistical change in the F1 generation (*P* > 0.05, Fig. 1C).

**Figure 2:**
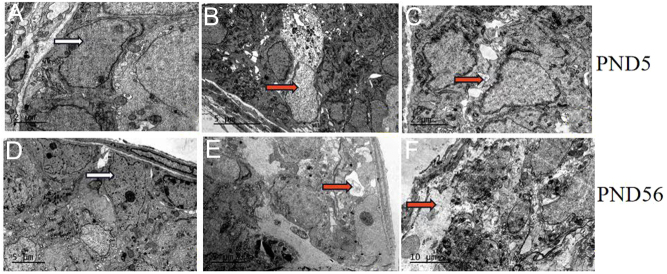
Effect of Cd exposure on the ultrastructure of testis of PND 5 and PND 56 of F1. A and D: Representative images from the control group. No observed lesions were found in the cell morphology. B, C, E and F: Representative images from the 2 mg/kg Cd-treated group. Oedema, necrosis (B: red arrow), enlarged space between surrounding cells (C: red arrow), cracking, vacuole structure (E: red arrow), atrophy and necrosis (F: red arrow) in Sertoli cells are observed. White arrow indicates normal morphology. *n* = 2 rats per group.

At the same time, on TEM examination, in the 2 mg CdCl_2_/kg-treated group, Sertoli cells showed different changes of ultrastructure compared to the control group. As shown in Fig. 2, at PND 5, in the control group, the nuclear membrane of the Sertoli cells was complete and the cell boundaries were clear, and morphology of the organelles were normal (white arrow in Fig. 2A). However, the 2mg/kg Cd-treated group showed obvious cell damage. Sertoli cells appeared necrotic, vacuolated (red arrow in Fig. 2B), nuclear density was reduced and the connection between Sertoli cells and spermatogenic cells was loose (red arrow in Fig. 2C). At PND 56, in the control group, the nuclear membrane of the Sertoli cells was complete and the cell boundaries were clear, and morphology of the organelles were normal (white arrow in Fig. 2D). However, the 2 mg/kg Cd-treated group showed obvious damage; Sertoli cells appeared lysed and vacuolated, atrophy and necrosis were evident, matrix density decreased, cell membrane disappeared and connections with surrounding cells were loose or even some cells peeled off the basal surface (red arrow in Fig. 2E and F). These changes indicated that the testicular tissue structure was abnormal after prenatal Cd exposure.

**Figure 3:**
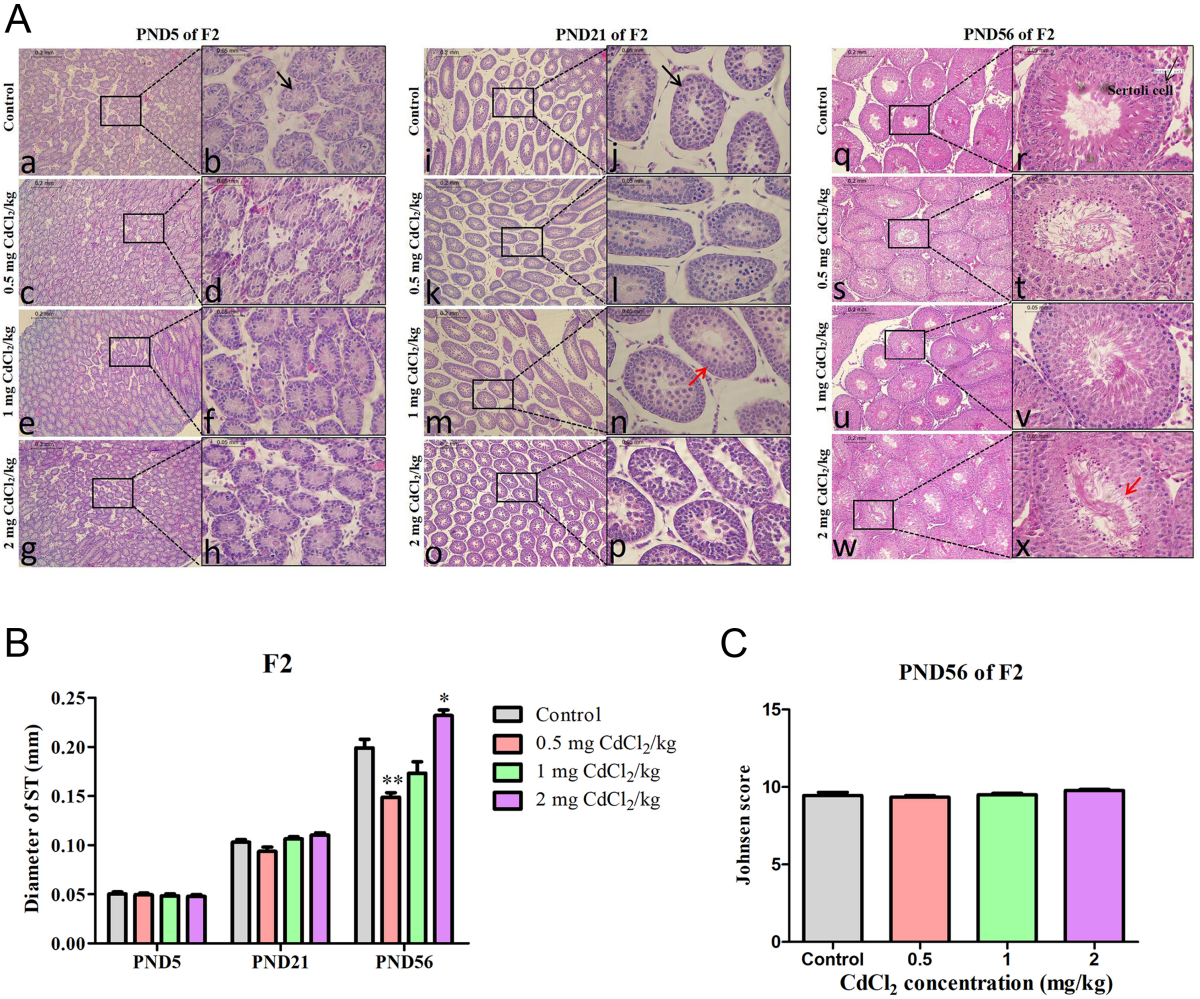
Effect of prenatal Cd exposure on testicular histology in the F2 generation rats. A: H&E staining at different stages of F2 generation after exposure to Cd during pregnancy. B: The tubular diameter (ST diameter of 375 tubules) of F2. C: The Johnsen score of PND 56 of F2. *n* = 5 rats per group. Data are presented as mean ± s.d., *P < 0.05 and **P < 0.01 compared with the control group. A black arrow means normal Sertoli cell.

In the F2 generation, at PND 5 (black arrow in Fig. 3Ab), 21 (black arrow in Fig. 3Aj) and 56 (black arrow in Fig. 3Ar), the histological performance of the ST was the same as that of the F1 generation in the control group.

However, some changes were shown at PND 56 which was different from F1 generation. In F2 generation, the diameter of ST in the 0.5 mg CdCl_2_/kg-treated group significantly decreased (*P* < 0.01), whilst the ST diameter significantly increased in the 2 mg CdCl_2_/kg-treated group (Fig. 3B, *P* < 0.05). Compared with the control group, the Johnsen score also had no statistical change in the F2 generation (*P* > 0.05, Fig. 3C).

### Prenatal Cd exposure interrupted steroid hormones in F1 and F2 generations

**Figure 4:**
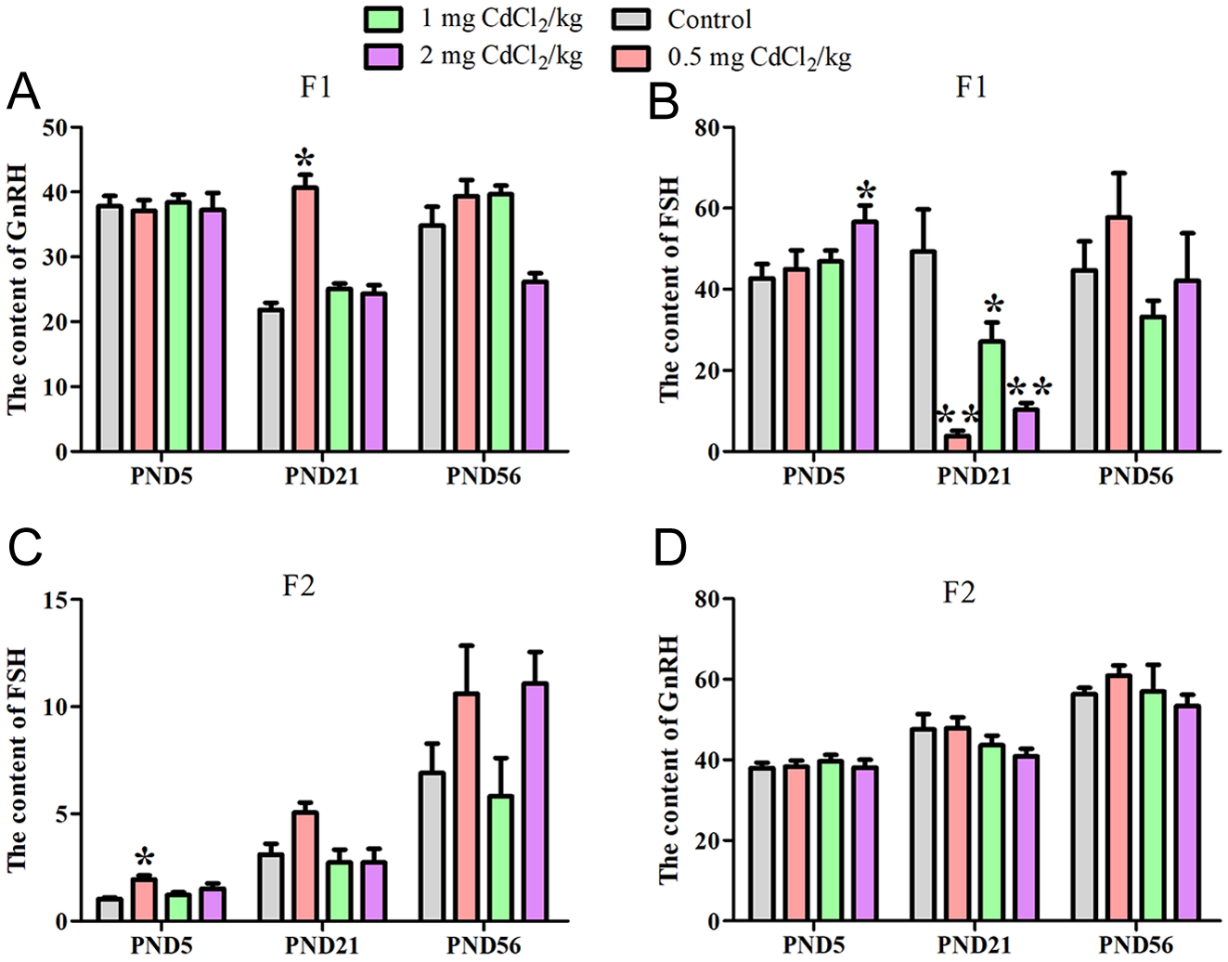
Effect of prenatal Cd exposure on steroid hormones in the male offspring rats. A: The levels of gonadotropin-releasing hormone (GnRH) in the serum of F1. B: The levels of the follicular-stimulating hormone (FSH) in the serum of F1. C: The levels of gonadotropin-releasing hormone (GnRH) in the serum of F2. D: The levels of the follicular-stimulating hormone (FSH) in the serum of F2. *n* = 9 rats per group. Data are presented as mean ± s.d., *P < 0.05 and **P < 0.01 compared with the control group.

In the F1 generation, at PND 5 (Fig. 4), compared with the control group, the GnRH levels in each Cd-treated group had no change (*P* > 0.05, Fig. 4A), and the FSH level of the 2 mg CdCl_2_/kg-treated group (56.60 pg/mL) was significantly up-regulated compared to that of the control group (42.68 pg/mL) (*P* < 0.05, Fig. 4B). At PND 21, compared with the control group, the GnRH level of the 0.5 mg CdCl_2_/kg-treated group was significantly up-regulated (*P* < 0.05, Fig. 4A), and the FSH level was also significantly down-regulated in all the treatment groups, with the lowest of 44.96 pg/mL in the 0.5 mg CdCl_2_/kg-treated group (*P* < 0.01, Fig. 4B). However, at PND 56, compared with the control group, the GnRH and FSH levels in each Cd-treated group had no change (*P* > 0.05, Fig. 4A and B).

In the F2 generation, the changes were only found in the FSH concentration at PND 5, compared with the control group, the FSH level in the 0.5 mg CdCl_2_/kg-treated group was significantly up-regulated compared to the control group (*P* < 0.05, Fig. 4D). At PND 21 and 56, the GnRH and FSH levels in each Cd-treated group had no change (*P* > 0.05, Fig. 4C and D).

### Prenatal Cd exposure decreased FSHR protein in Sertoli cells in the F1 generation

**Figure 5:**
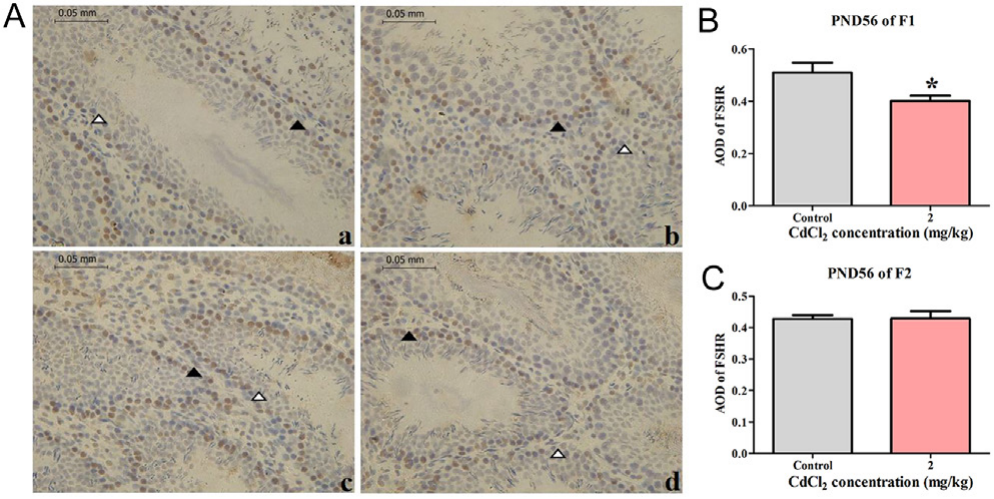
Effect of prenatal Cd exposure on FSHR protein in Sertoli cells in the male offspring rats. A: The FSHR protein in Sertoli cells in F1 and F2 (positive cells shown with the black triangle; negative cells shown with the white triangle). a and c: The FSHR protein in Sertoli cells in the control group. b and d: The FSHR protein in Sertoli cells in 2 mg CdCl_2_/kg-treated group. a and b: The FSHR protein in Sertoli cells in PND 56 of F1. c and d: The FSHR protein in Sertoli cells in PND 56 of F2. B: The FSHR protein expression in Sertoli cells in PND 56 of F1. C: The FSHR protein expression in Sertoli cells in PND 56 of F2.* n* = 5 rats per group. Data are presented as mean ± s.d., *P < 0.05 compared with the control group.

In the F1 and F2 generations, as shown in Fig. 5A, Sertoli cells were distributed in the basement membrane of ST, and no Sertoli cells peeled off the base surface in the control group (Fig. 5Aa and c). However, after staining with FSHR, positive (black triangle) and negative (white triangle) Sertoli cells and the AOD value of FSHR-positive cells in 2 mg CdCl_2_/kg-treated group significantly decreased compared to the control group (*P* < 0.05, Fig. 5B) in the F1 generation at PND 56. However, the AOD value of FSHR-positive cells in the 2 mg CdCl_2_/kg-treated group had no change compared with the control group in the F2 generation (Fig. 5C).

### Prenatal Cd exposure changed the FSHR/PI3K/AKT/FOXO1 signalling pathway in the F1 and F2 generations

#### The mRNA levels of FSHR/PI3K/AKT/FOXO1 factors changed in the F1 and F2 generations

**Figure 6:**
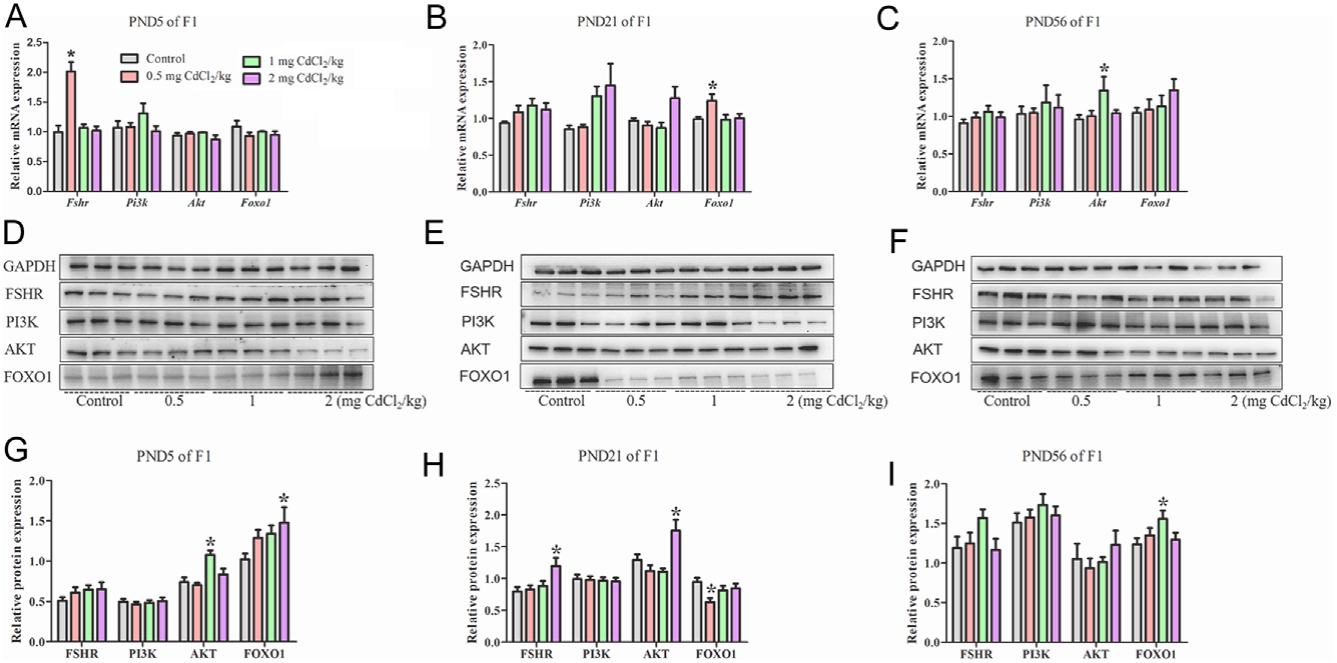
Effects of prenatal Cd exposure on FSHR/PI3K/AKT/FOXO1 signalling pathway in the F1 generation rats. A, B and C: The relative mRNA repression of FSHR/PI3K/AKT/FOXO1 pathway. D, E and F: Protein band chart of FSHR/PI3K/AKT/FOXO1 pathway. G, H and I: The protein repression of FSHR/PI3K/AKT/FOXO1 pathway. *n* = 5 rats per group. Data are presented as mean ± s.d.,*P < 0.05 compared with the control group.

In the F1 generation, compared with the control group, only the *Fshr* mRNA expression in 0.5 mg CdCl_2_/kg-treated group was significantly increased at PND 5 (Fig. 6A, *P* < 0.05). At PND 21, the* Foxo1* mRNA expression in the 0.5 mg CdCl_2_/kg-treated group was significantly increased in Fig. 6B (*P* < 0.05). At PND 56, only the* Akt* mRNA expression in the 1 mg CdCl_2_/kg-treated group was significantly increased in Fig. 6C (*P* < 0.05). The changes in the expression of these genes in the F1 generation at different stages have no continuity.

**Figure 7:**
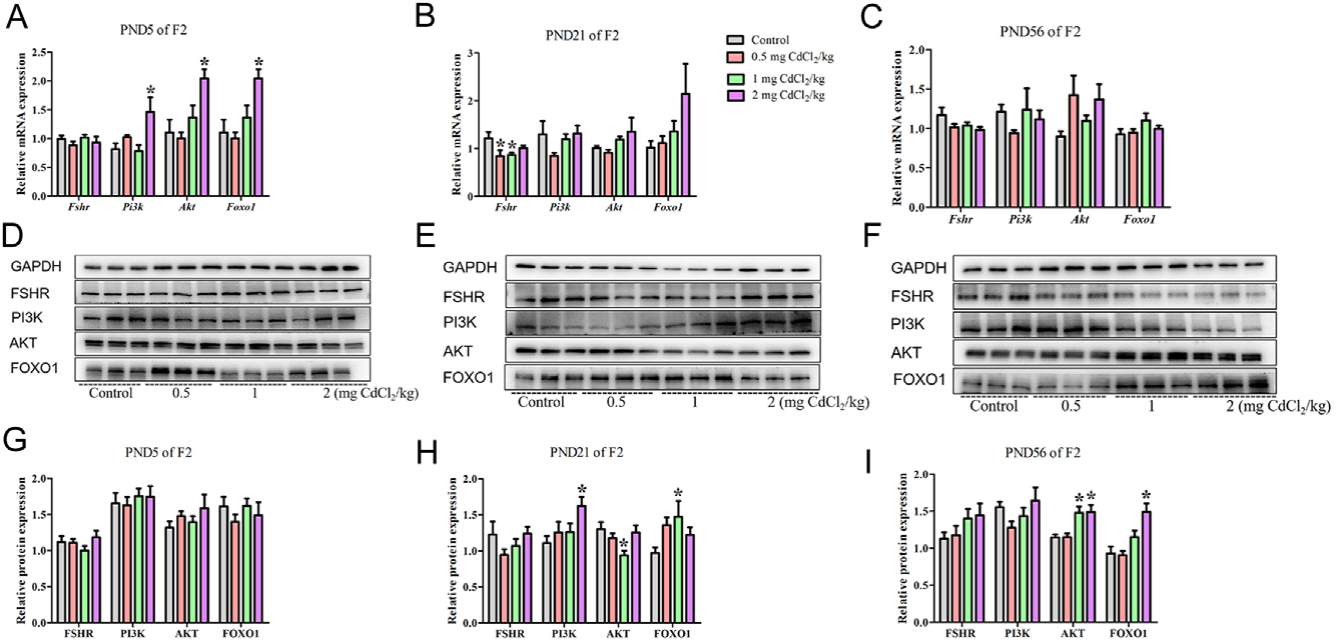
Effects of prenatal Cd exposure on FSHR/PI3K/AKT/FOXO1 pathway in the F2 generation rats. A, B and C: The relative mRNA repression of FSHR/PI3K/AKT/FOXO1 pathway. D, E and F: Protein band chart of FSHR/PI3K/AKT/FOXO1 pathway. G, H and I: The protein repression of FSHR/PI3K/AKT/FOXO1 pathway. *N* = 5 rats per group. Data are presented as mean ± s.d., *P < 0.05 compared with the control group.

In the F2 generation, compared with the control group, the *Pi3k, Akt* and *Foxo1* mRNA expression in the 2 mg CdCl_2_/kg-treated group was significantly increased at PND 5 (Fig. 7A, *P* < 0.05). At PND 21, the *Fshr* mRNA expression in 0.5 and 1 mg CdCl_2_/kg-treated group was significantly decreased in Fig. 7B (*P* < 0.05). However, at PND 56, there was no significant change in mRNA expression among the groups in Fig. 7C (*P* > 0.05). The changes of these genes in the F2 generation at different stages also have no continuity and the changes were different from the F1 generation.

#### The protein levels of FSHR/PI3K/AKT/FOXO1 factors changed in F1 and F2 generations

In the F1 generation, compared with the control group, at PND 5, the AKT protein expression in 1 mg CdCl_2_/kg-treated group and the FOXO1 protein expression in 2 mg CdCl_2_/kg-treated group were significantly increased in Fig. 6G (*P* < 0.05). At PND 21, the FOXO1 protein expression was significantly decreased, and the FSHR and AKT protein expression in the 2 mg CdCl_2_/kg-treated group were significantly increased in Fig. 6H (*P* < 0.05). At PND 56, only the FOXO1 protein expression in the 1 mg CdCl_2_/kg-treated group was significantly increased in Fig. 6I (*P* < 0.05). Only FOXO1 protein changed at different stages but increased at PND 5 and 56 and decreased at PND 21.

In the F2 generation, compared with the control group, at PND 5, there was no significant change in protein expression among the groups in Fig. 7G (*P* > 0.05). At PND 21, the PI3K protein expression in the 2 mg CdCl_2_/kg-treated group was significantly increased (Fig. 7H, *P* < 0.05). The AKT protein expression in the 1 mg CdCl_2_/kg-treated group was significantly decreased (*P* < 0.05), but the FOXO1 protein expression in 1 mg CdCl_2_/kg-treated group was significantly increased in Fig. 7H (*P* < 0.05). At PND 56, the AKT protein expression in the 1 and 2 mg CdCl_2_/kg-treated group was significantly increased, and the FOXO1 protein expression in 2 mg CdCl_2_/kg-treated group was significantly increased in Fig. 7I (*P* < 0.05). FOXO1 protein increased in both PND 21 and 56, and AKT protein decreased first and then increased in the F2 generation. FOXO1 protein changed both in F1 and F2 generations with different patterns.

#### Several sites of DNA methylation levels in the promoter regions of fshr/akt/foxo1 genes changed in the F1 and F2 generations

At PND 21 and 56, the promoter of DNA methylation in the F1 and F2 generations were detected. Compared with the control group, the general DNA methylation of *Fshr*, *Akt* and *Foxo1* genes had no change (*P* > 0.05) (Supplementary Fig. 2).

**Figure 8:**
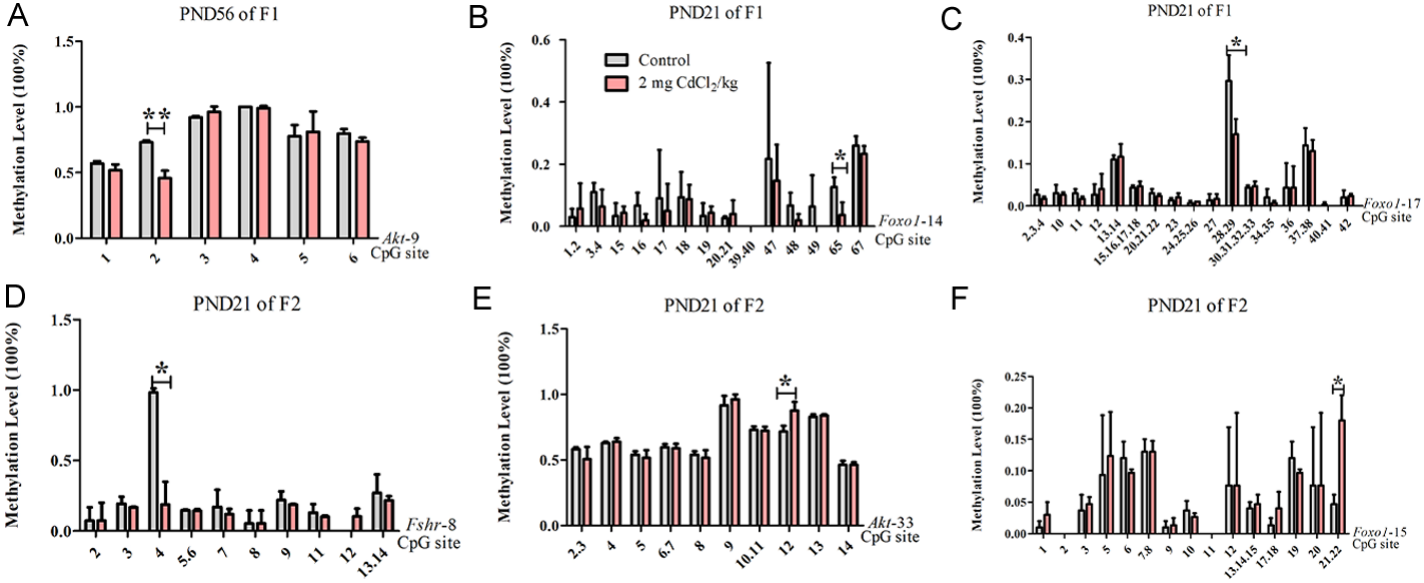
Effects of prenatal Cd exposure on DNA methylation levels in the promoter regions of *Fshr/Akt/Foxo1* genes in the male offspring rats. A–C: The methylation level of *Fshr, Akt* and *Foxo1* gene relative promoter region of F1. D–F: The methylation level of *Fshr, Akt* and *Foxo1* gene relative promoter region of F2. *n* = 3 rats per group. Data are presented as mean ± s.d., *P < 0.05 and **P < 0.01 compared with the control group.

At PND 56, in the F1 generation, the methylation level of *Akt-9* fragment significantly decreased at the second position site (*P* < 0.01, Fig. 8A).

At PND 21, in the F1 generation, the methylation levels significantly decreased at position 65 sites of the *Foxo1-14* fragment (*P* < 0.05, Fig. 8B), position 28 and 29 sites of the *Foxo1-17* fragment (*P* < 0.05, Fig. 8C).

At PND 21, in the F2 generation, the methylation level of the Cd-treated group significantly decreased at the fourth position site of the *Fshr-8* fragment (*P* < 0.05, Fig. 8D), the methylation level of the Cd-treated group significantly decreased at the 12th position site of the *Akt-33* fragment (*P* < 0.05, Fig. 8E) and the methylation level of the Cd-treated group significantly increased at the 21st and 22nd sites of the *Foxo1-15* fragment (*P* < 0.05, Fig. 8F).

### Prenatal Cd exposure changed DNA methyltransferase in the male offspring rats

#### The mRNA levels of DNA methyltransferase changed in F1 and F2 generations

**Figure 9:**
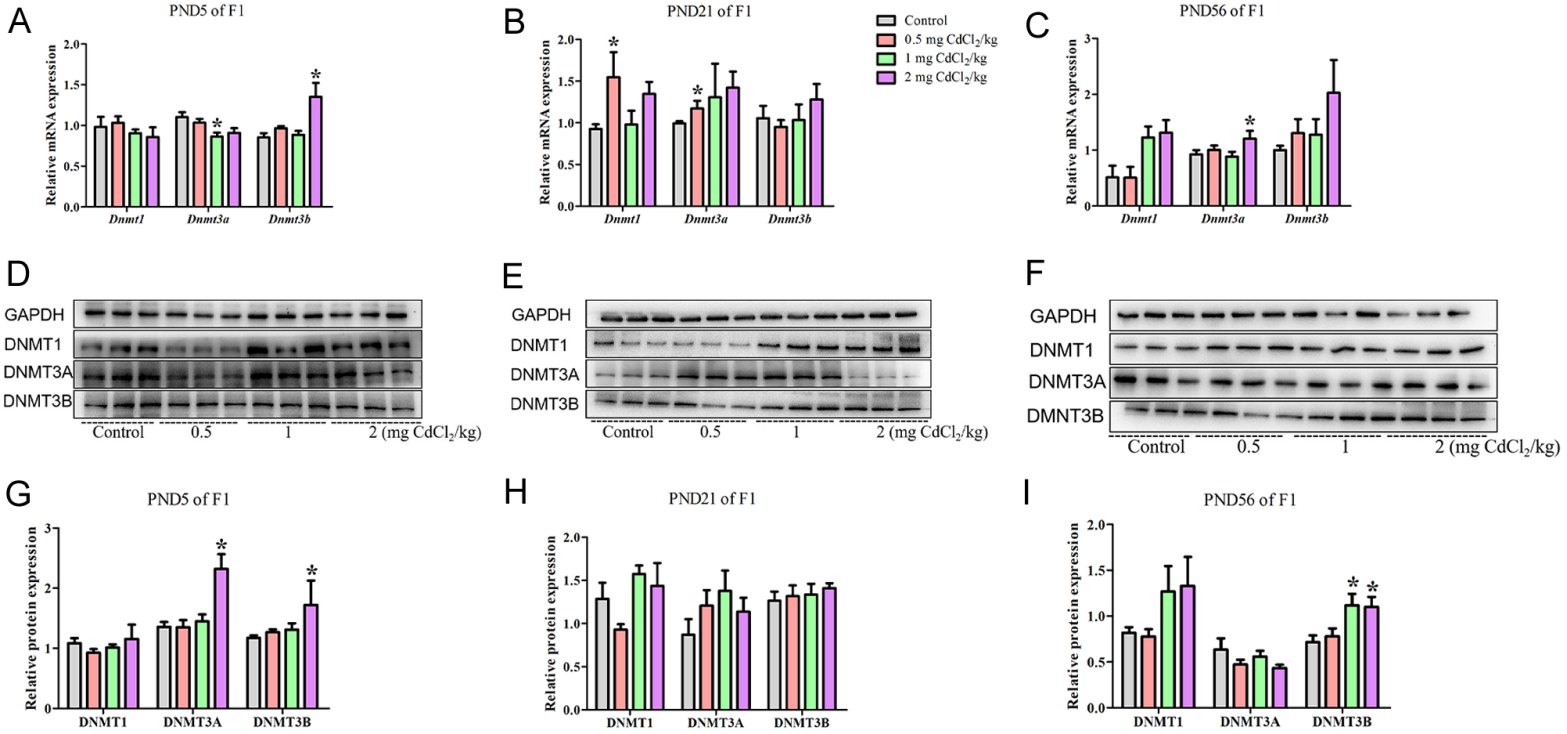
Effects of prenatal Cd exposure on DNA methyltransferase in the F1 generation rats. A, B and C: The relative mRNA repression of DNA methyltransferase. D, E and F: Protein band chart of DNA methyltransferase. G, H and I: The protein repression of DNA methyltransferase. *n* = 5 rats per group. Data are presented as mean ± s.d., *P < 0.05 compared with the control group.

In the F1 generation, compared with the control group, at PND 5, per the mRNA analysis results shown in Fig. 9A, the* Dnmt3a* mRNA expression in the 1 mg CdCl_2_/kg-treated group was significantly decreased (*P* < 0.05). At PND 21, per the mRNA analysis results shown in Fig. 9B, the *Dnmt1* and *Dnmt3a* mRNA expression in the 0.5 mg CdCl_2_/kg-treated group were significantly increased (*P* < 0.05). At PND 56, per the mRNA analysis results shown in Fig. 9C, the *Dnmt3a* mRNA expression in the 2 mg CdCl_2_/kg-treated group was significantly increased (*P* < 0.05). *Dnmt3a* mRNA expression in F1 generation decreased first and then decreased at PND 5, then increased again at PND 21 and 56.

**Figure 10:**
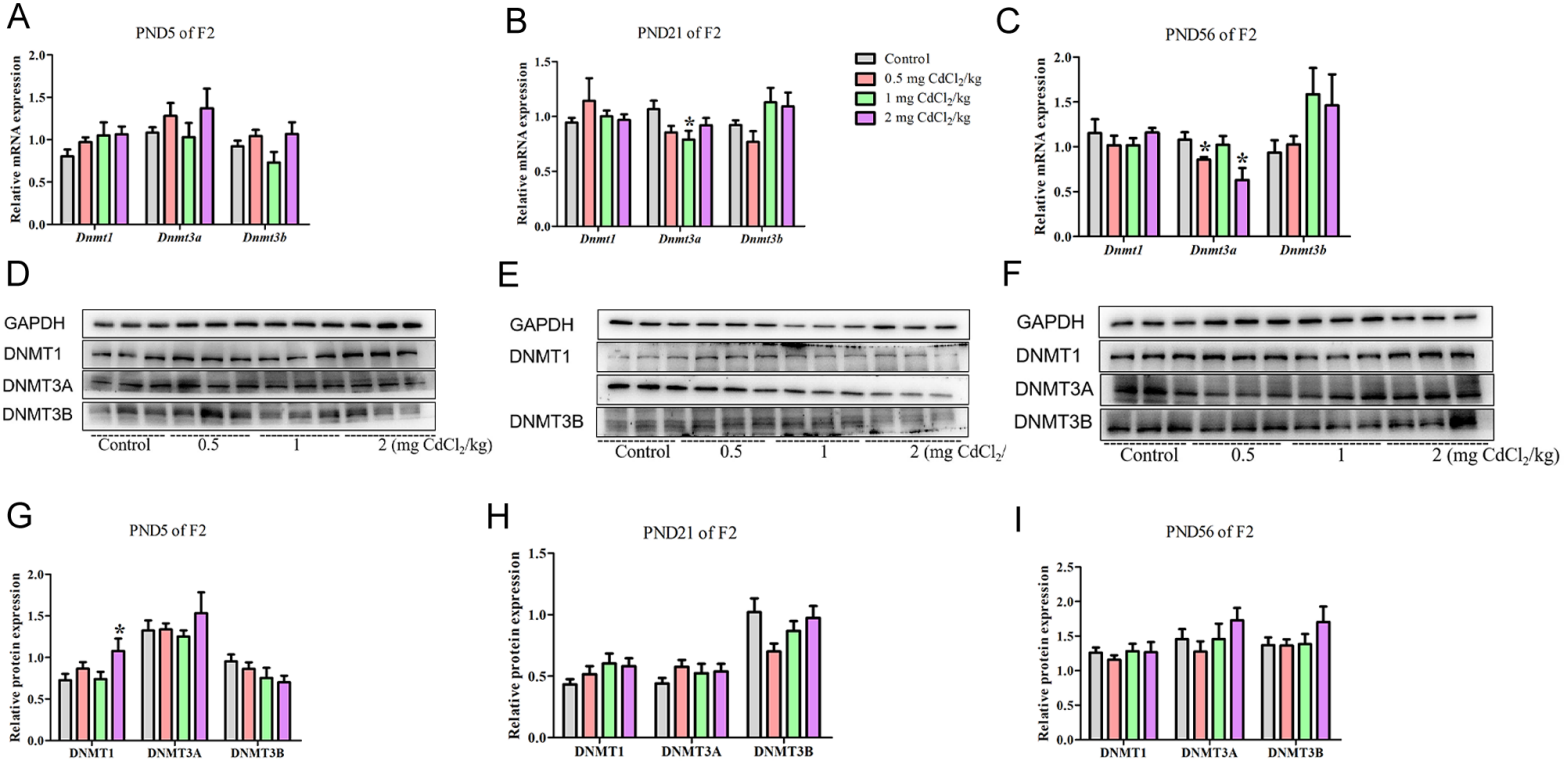
Effects of prenatal Cd exposure on DNA methyltransferase in the F2 generation rats. A, B and C: The relative mRNA repression of DNA methyltransferase. D, E and F: Protein band chart of DNA methyltransferase. G, H and I: The protein repression of DNA methyltransferase. *n* = 5 rats per group. Data are presented as mean ± s.d., *P < 0.05 and **P < 0.01 compared with the control group.

In the F2 generation, compared with the control group, at PND 5, per the mRNA analysis results shown in Fig. 10A, there was no statistical difference in mRNA expression among these genes (*P* > 0.05). At PND 21, per the mRNA analysis results shown in Fig. 10B, the *Dnmt3a* mRNA expression in the 1 mg CdCl_2_/kg-treated group was significantly decreased (*P* < 0.05). At PND 56, per the mRNA analysis results shown in Fig. 10C, the *Dnmt3a* mRNA expression in 0.5 and 2 mg CdCl_2_/kg-treated groups was significantly decreased (*P* < 0.05). *Dnmt3a* mRNA expression in the F2 generation decreased at both PND 21 and 56, however, pattern of change was different in F1 and F2 generations.

#### The protein levels of DNA methyltransferase changed in F1 and F2 generations

In the F1 generation, compared with the control group, at PND 5, per the protein results shown in Fig. 9G, the DNMT3A and DNMT3B protein expression in the 2 mg CdCl_2_/kg-treated group were significantly increased (*P* < 0.05). At PND 21, as shown in Fig. 9H, there was no significant change in protein expression among the groups for these proteins (*P* > 0.05). At PND 56, as shown in Fig. 9I, the DNMT3B protein expression in 1 and 2 mg CdCl_2_/kg-treated groups was significantly increased (*P* < 0.05). At these three stages of the F1 generation, DNMT3B protein increased both at PND 5 and 56.

In the F2 generation, at PND 5, per the protein results shown in Fig. 10G, compared with the control group, the DNMT1 protein expression in the 2 mg CdCl_2_/kg-treated group was significantly increased (*P* < 0.05). At PND 21, as shown in Fig. 10H, there was no significant change in protein expression among the groups for these proteins (*P* > 0.05). At PND 56, as shown in Fig. 10I, there was also no statistical change in protein expression among the groups (*P* > 0.05). At these three stages of the F2 generation, only DNMT1 protein expression changed at PND 5. Among these proteins, F1 and F2 generations have different patterns.

## Discussion

In this study, we first report the inter-generational effect of Cd on Sertoli cells and provide a new insight into the inter-generational mechanisms of Cd-induced testicular injury. Our findings demonstrate an inter-generational adverse effect of Cd on male reproduction and highlight the generation-specific heritable effects of Cd.

Our previous study showed that prenatal Cd resulted in serum ovarian, testicular injury and hormone disorder (T, P4 and E2) in the F1 and F2 generations after mating treated females (from F0 generation) with untreated males (Huang *et al.* 2020, Liu *et al.* 2020). In this study, we found that Cd also causes multigenerational testicular injury in another animal model, where treated males (from F0 generation) were mated with untreated females. The results showed that the Johnson score did not change in the F1 and F2 generations at postnatal day 56 (PND 56); however, the ST morphology was impaired at different developmental stages. The F1 generation showed reduced spermatogenesis characterised by smaller ST diameters, and the F2 generation showed only one spermatogenic cell layer in some STs. We also found that Sertoli cells have severe damage in the F1 and F2 generations after Cd exposure during pregnancy. The number of Sertoli cells decreased in the F1 generation and they appeared lysed and vacuolated, atrophy and necrosis were evident, matrix density decreased, cell membrane disappeared and connections with surrounding cells were loose or some cells peeled off the basal surface; all these changes in Sertoli cells were observed both at PND 5 and 56 with different degrees of damage. Therefore, Cd exposure during pregnancy can affect the tight connections of cells in STs; testicular injury also happened at different stages as Sertoli cells may be one of the main targets for Cd.

Further analyses of the mechanism of Cd on Sertoli cells showed that abnormal expression of FSH in different growth stages could be one of the causes. As an environmental endocrine disruptor, Cd has been shown to interfere with hormone secretion and affect the hypothalamic–pituitary–hormone axis (Zhang *et al.* 2011, Ji *et al.* 2015). Our results showed that Cd exposure altered FSH levels in both the F1 and F2 generations and interfered with GnRH levels. However, there was a different trend in the levels of serum FSH and GnRH between the F1 and F2 generations as the F2 generation could have made corresponding adjustments after the F1 injury caused by prenatal Cd exposure. Our previous research also found a significant decrease in serum testosterone (TS) in F1 rats, while a significant increase in serum TS was observed in F2 rats after prenatal exposure to Cd in another animal model (Huang *et al.* 2020), which may be due to corresponding adjustments being made between different generations. In the present study, cyclical changes with a bell trend in FSH activity were observed in the control group at three different developmental stages (PND 5, 21 and 56), but for the treatment groups, the data showed U trend in F1 generation. Especially at PND 21, compared to control groups, the level of FSH significantly decreased in all treatment groups. After prenatal Cd exposure, the male rats had a different pattern of FSH activity in the F1 generation, which may be induced by testicular injury. In the meantime, we also found that the FSH trend showed a different pattern in the F2 generation from the F1 generation; the control group and treatment groups showed the same linear trend with growth stages, and even no statistics changed between the control group and treatment groups. The change in FSH secretion between F1 and F2 generations may be an important cause of Sertoli cell damage after prenatal Cd exposure. In addition, the data also suggests that the decline in FSH levels in the F1 generation at PND 21 may be related to the level of GnRH being up-regulated after prenatal Cd exposure.

On the other hand, as a target of Cd on testicular cells, Sertoli cells can express FSHR, which is a marker for these cells; our study found that prenatal Cd exposure decreased the number of Sertoli cells in the testes of rats, as indicated by a decreased area of density (AOD) value of the FSHR protein in the F1 generation. Prenatal Cd exposure may affect the number of Sertoli cells in the F1 generation. Research has shown that changes in the number of Sertoli cells can affect the number of spermatogenic cells and the size of the testis (Pfaff *et al.* 2013). Our study also found after prenatal Cd exposure, the diameter of ST decreased in the F1 generation. These findings also suggested that prenatal Cd exposure can cause testicular injury by targeting Sertoli cells in offspring.

Furthermore, research also found that FSH primarily performs its role by binding to the FSHR (Urrutia *et al.* 2019), and after this binding, they can activate FSHR/PI3K/AKT/FOXO1 pathway to affect Sertoli cell proliferation; this pathway may be a molecular target by chemicals and ultimately cause the shedding of spermatogenesis cells (Chen *et al.* 2017). Our results also showed that Cd exposure may interfere with the FSHR/PI3K/AKT/FOXO1 pathway and then cause changes in Sertoli cells. In the F1 generation, the mRNA and protein levels of these genes had a different pattern at different stages: for FSHR, the mRNA level increased at PND 5, and the protein level increased at PND 21; for AKT, the mRNA level increased at PND 56, and protein level increased at PND 21 and 56; for FOXO1, the mRNA level increased at PND 21, and protein level increased at PND 5 and56 but decreased at PND 21; none of PI3K changed. Based on the data, we find that prenatal Cd exposure may increase FSHR/PI3K/AKT/FOXO1 pathway protein, and results in Sertoli cells proliferation at PND 56, however, the immunohistochemistry results showed that Sertoli cells decrease, which means other factors may also be involved and more studies are required in the future. In the F2 generation, the mRNA levels of *Pi3k*, *Akt* and *Foxo1* increased at PND 5, but *Fshr* decreased at PND 21; the protein levels of PI3K, AKT and FOXO1 increased at PND 56, but AKT decreased at PND 21.

Taken together, our data show that changes in the protein levels of these factors were not fully consistent with changes in their mRNA expression levels at different doses and developmental stages. The changes were not entirely consistent between F1 and F2 generations. In our previous studies, we also found that prenatal Cd exposure can alter factors with different patterns in multiple generations (Huang *et al.* 2020, Liu *et al.* 2020). The reasons for the differences of these genes’ expression between F1 and F2 generations may be related to the prenatal Cd exposure. For the F1 generation, the germ cells may be affected by the direct exposure of the rat fetus to Cd l in the uterus; however, the rats in the F2 generation were not directly exposed to Cd. Meanwhile, the reason the mRNA and protein expression of the same factor was inconsistent is because the gene expression does not strictly follow the rules of the centre. The rules of the centre refers to the process of transmitting genetic information from DNA to RNA, and then from RNA to protein, completing the transcription and translation of genetic information. Our study shows that greater the mRNA produced by DNA transcription, theoretically there should be more subsequent protein synthesis and higher expression levels. The process of mRNA transcription produces proteins, and the transcription of that adjustment process is influenced by Cd. However, our results revealed that the effect of prenatal Cd exposure did not change the promoter of CpG islands in DNA methylation in the F1 and F2 generations at either PND 21 or PND56. However, methylation at some sites of *Foxo1* and *Akt* decreased, and genes still have a stres effect on the toxic effects of Cd. At all events, the results indicate that prenatal Cd can interfere with the FSHR/PI3K/AKT/FOXO1 signalling pathway and further study is needed. It has also been indicated that the effects of Cd exposure during pregnancy on this signalling pathway are multigenerational.

Epigenetic modification may be another important mechanism of reproductive damage caused by Cd (Eliveld *et al.* 2019). Our previous data showed that after prenatal exposure to Cd, a large number of individual CpG sites throughout the genome changed in rat testes in the F1 generation (559 up-regulated and 171 down-regulated), suggesting that DNA methylation may be involved in the Cd-related impairment of testicular injury. Interestingly, our data also found that the Cd-induced changes in DNA methylation correlated with altered levels of expression of DNA methyltransferase in offspring. Similar to other studies, DNMT3 was the main target of Cd. In the F1 generation, the protein levels of DNMT3A and DNMT3B were up-regulated at PND 5 and PND56 with the concentration increasing, but the mRNA levels were down-regulated at PND 5 at 1 mg CdCl_2_/kg-treated group and up-regulated at PND 21 and PND56. In the F2 generation, the mRNA level of *Dnmt3a* was also down-regulated at PND 21 and PND56 in 0.5 or 2 mg CdCl_2_/kg-treated groups. Cd can cause a monotonic dose–response relationship, for example the protein levels DNMT3B in the F1 generation at PND 56 increases with increasind dosage, however, the effect of Cd on DNA methyltransferase was not simply a monotonic dose-response relationship, Cd also caused nonmonotonic dose responses in this study. For example, the mRNA level of *Dnmt3a* has a nonlinear effect of prenatal Cd exposure in F2 male offspring at PND 56 in 0.5 mg treated group as it decreased with an increase in dosage. A nonmonotonic dose response is typical among common endocrine-disrupting chemicals, although the mechanisms behind such nonmonotonic effects are not fully understood; it is likely due to the hormetic effect of Cd on the activity of enzymes at different concentrations. Cd-induced changes in the levels of DNA methyltransferases could be one mechanism underlying the epigenetic changes caused by the prenatal Cd exposure. Moreover, our DNA methylation of promoter region analysis also found some interesting connections for *Foxo1* and *Akt* genes which may be caused by changes in DNA methyltransferases.

As a further aspect important to the study, our data first revealed that prenatal Cd exposure has an inter-generational effect on testicular injury. At the same time, in this study, we mainly used the paternal model. After prenatal exposure (F0 generation), the male rats exposed in the F1 generation mated with the normal female rats to produce F2-generation male rats. Therefore, testicular tissue damage occurred in the male rats in the F1 generation after intrauterine Cd exposure. Subsequently, the Y chromosome in the F2 generation came from male rats in the F1 generation that were exposed to Cd. Therefore, Cd exposure during pregnancy may cause reproductive problems in offspring male rats by affecting the Y chromosome, and this inter-generational genetic effect of Cd can be partly attributed to the paternal genetic effect, or it may also be due to the influence of epigenetic mechanisms, which warrants more research. At the same time, our data found that the expression of AKT, FOXO1 factors and FSHR protein was up-regulated. Combined with the results of F1 and F2 generations, it can be seen that Cd exposure during pregnancy can affect the expression of FSHR protein, which is also an upstream factor of this signalling pathway and may also be the target of Cd. By affecting the expression of FSHR, the signalling pathway was further affected, and finally, Sertoli cells were damaged. However, the validation of this target requires further experimental studies, such as Cd exposure after Sertoli cell knockout of FSHR, to observe the changes in signalling pathways and Sertoli cells. In addition, the results also showed that the expression of DNMT1 and DNMT3B increased, but DNMT3A decreased. Some previous studies have found that over-expression of DNMT3B can lead to hypermethylation and gene silencing in gene-specific promoter regions (Subramaniam *et al.* 2014). Thus, the inter-generational epigenetic inheritance of Cd exposure during pregnancy affects the whole-genome methylation level of the testis by promoting DNMT3B over-expression and then damaging the offspring testis.

In conclusion, prenatal Cd exposure can damage the testicular tissue and Sertoli cell structure of offspring and affect the growth and development of the testis. The changes in different periods were not continuous but had inter-generational toxicity effects. Cd can interfere with the secretion of multigeneration serum hormones (GnRH, FSH) and inhibit the expression of Sertoli cell FSHR protein in the F1 generation. The primary possible mechanism is to regulate the expression of factors related to the FSHR/PI3K/AKT/FOXO1 pathway (Supplementary Fig. 3). Moreover, prenatal Cd exposure can affect the genomic DNA methylation level of offspring testis and affect the DNA methylation pattern in testis tissue by interfering with the expression of DNA methylation transferases (DNMT1, DNMT3A, DNMT3B) in each generation and stage, which has an inter-generational epigenetic inheritance effect.

## Supplementary Materials

Figure 1 Generation pattern diagram.

Figure 2 Effects of prenatal Cd exposure on DNA methylation levels in the promoter regions of Fshr/Akt/Foxo1 genes in the male offspring rats.

Figure 3 Full text conclusion chart.

## Declaration of interest

The authors declare that there is no conflict of interest that could be perceived as prejudicing the impartiality of the research reported.

## Funding

This work was supported by the Natural Science Foundation of Fujian Province (Grant No. 2019J01313, 2018Y9101, 2015J01299 and 2016J01359), the National Natural Science Foundation of China (Grant No. 81673212 and 81373027), and the Health Science and Family Planning Research Project in Fujian (Grant No. 2017-CX-36).

## Ethics approval

This experimental study passed the ethical review of animal medicine in China of contract number 2018-12DM093.

## Availability of data and materials

All relevant data would be made available on request.

## Author contribution statement

LXQ, LZL and DXS conceived the study and wrote the paper. YYB, ZJL and LYC plotted the pictures, collected the data and analysed the data. LJ and ZWC conceived, reviewed, organised, revised and also supported with the funding.
