# Supplementary figures and images for: Prenatal cadmium exposure has inter-generational adverse effects on Sertoli cells through the follicle-stimulating hormone receptor pathway
Source: Reproduction. 2023 Sep 4;166(4):271–84. doi: 10.1530/REP-23-0070 (PMC10502957; doi:10.1530/REP-23-0070)

F0

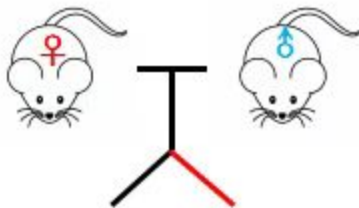

F1

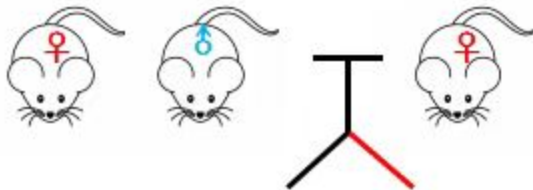

F2

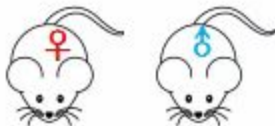

Supplement: Figure 1 Generation pattern diagram. [file supplementary_figure_1.pdf]

F1

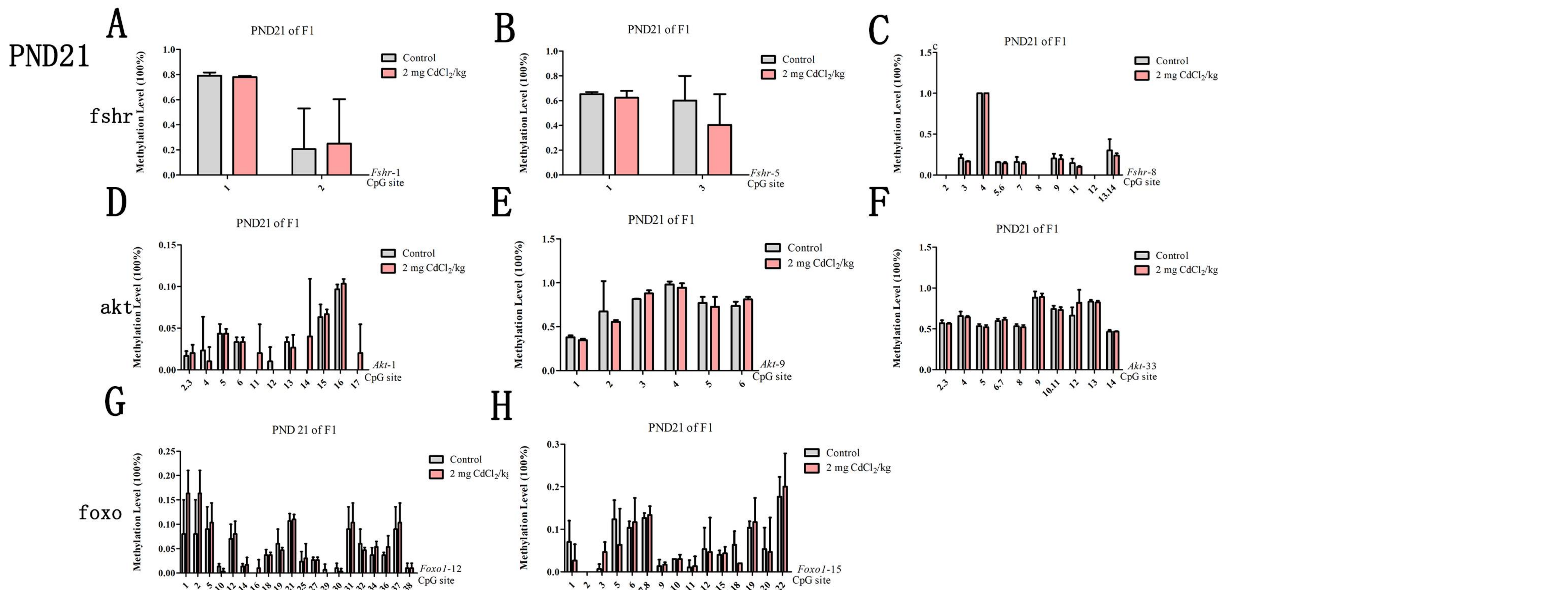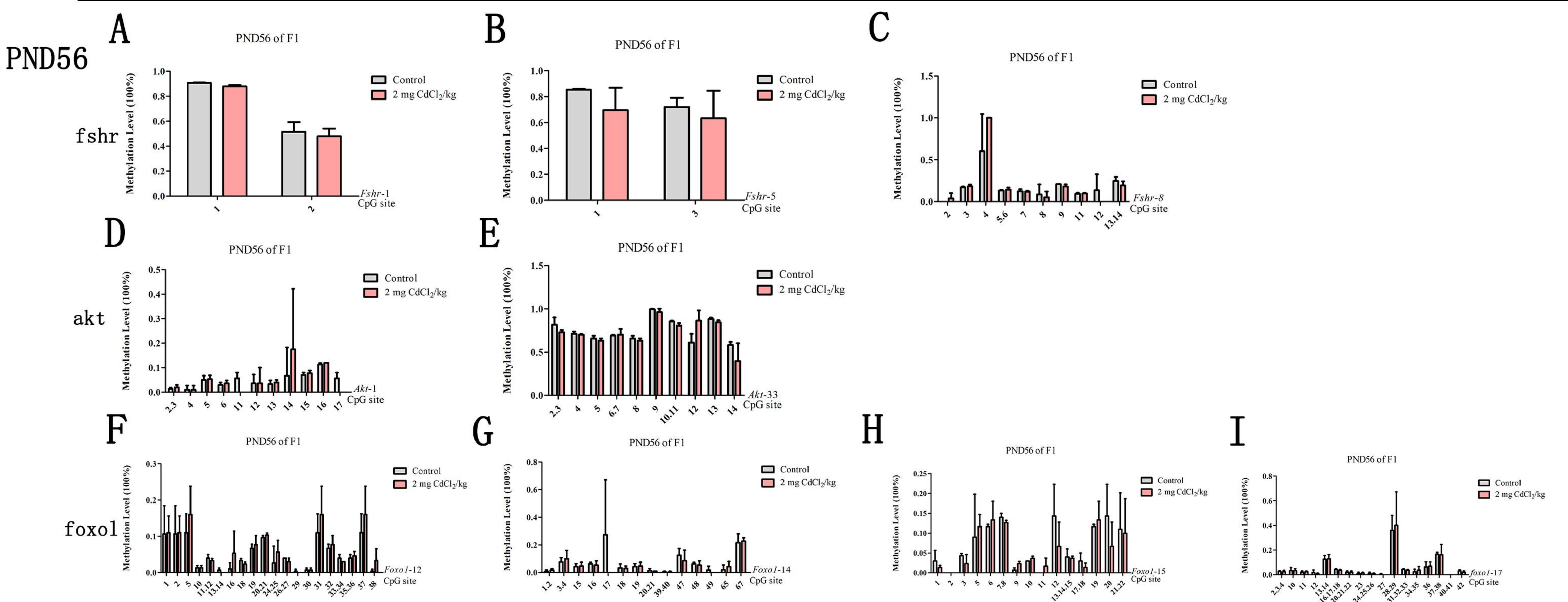

F2

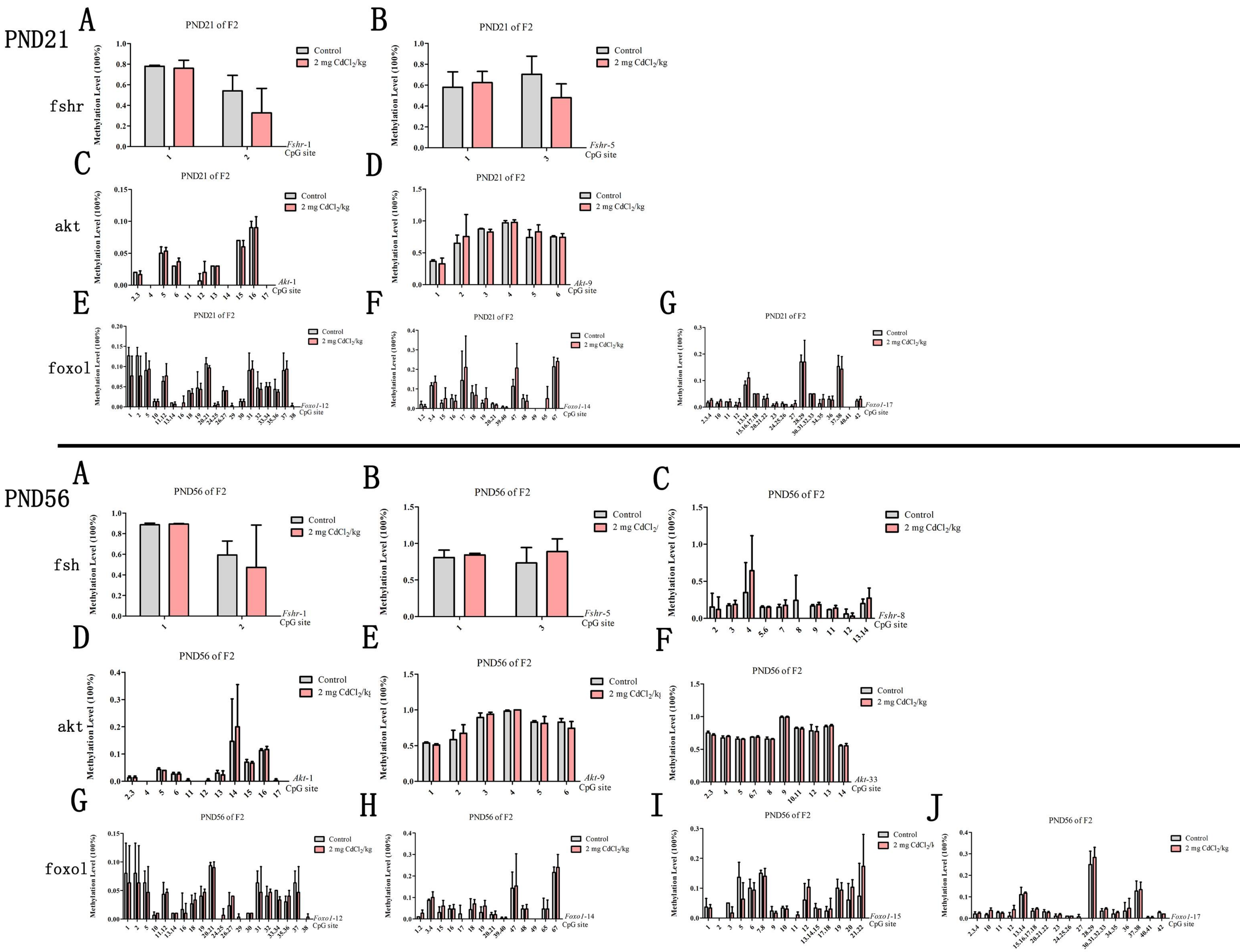

Supplement: Figure 2 Effects of prenatal Cd exposure on DNA methylation levels in the promoter regions of Fshr/Akt/Foxo1 genes in the male offspring rats. [file supplementary_figure_2.pdf]

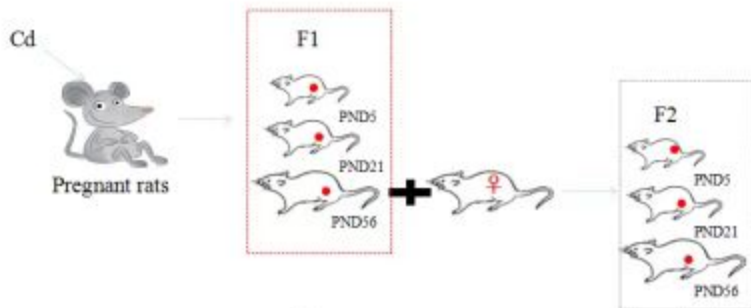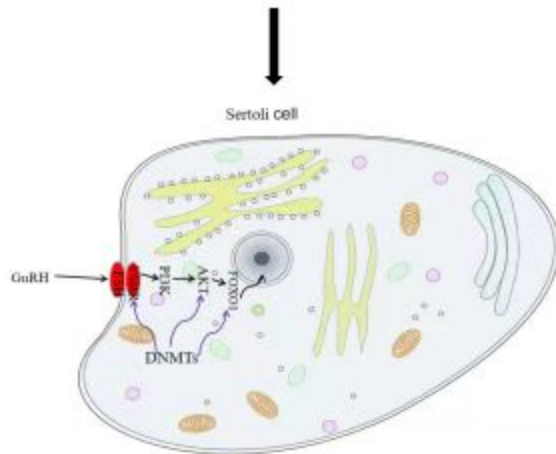

Testicular development damage

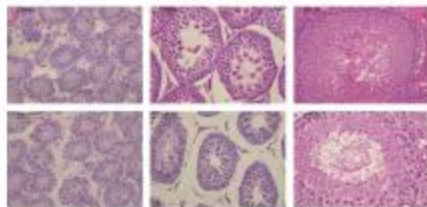

Serum

GnRH  
FSH

Sertoli cell locate

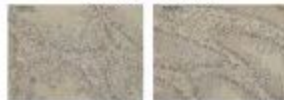

Supplement: Figure 3 Full text conclusion chart. [file supplementary_figure_3.pdf]
